# Capsanthin induces G1/S phase arrest, erlotinib-sensitivity and inhibits tumor progression by suppressing EZH2-mediated epigenetically silencing of p21 in triple-negative breast cancer cells

**DOI:** 10.18632/aging.202925

**Published:** 2021-05-02

**Authors:** Jia-Yan Wu, Yi-Chung Chien, I-Chen Tsai, Chih-Chiang Hung, Wei-Chien Huang, Liang-Chih Liu, Yung-Luen Yu

**Affiliations:** 1 Graduate Institute of Biomedical Sciences, China Medical University, Taichung 40402, Taiwan; 2 Ph.D. Program for Translational Medicine, China Medical University, Taichung 40402, Taiwan; 3 Institute of New Drug Development, China Medical University, Taichung 40402, Taiwan; 4 Drug Development Center, Research Center for Cancer Biology, China Medical University, Taichung 40402, Taiwan; 5 Center for Molecular Medicine, China Medical University Hospital, Taichung 40402, Taiwan; 6 Division of Breast Surgery, Department of Surgery, Taichung Veterans General Hospital, Taichung 40705, Taiwan; 7 Department of Applied Cosmetology, College of Human Science and Social Innovation, Hungkuang University, Taichung 43302, Taiwan; 8 School of Medicine, College of Medicine, China Medical University, Taichung 40402, Taiwan; 9 Department of Surgery, China Medical University Hospital, Taichung 40402, Taiwan; 10 Department of Medical Laboratory Science and Biotechnology, Asia University, Taichung 41354, Taiwan

**Keywords:** capsanthin, triple-negative breast cancer (TNBC), EZH2, p21, erlotinib

## Abstract

Capsanthin is a naturally occurring red pepper carotenoid with possible antitumor activity, but its antitumor mechanisms have yet to be delineated. We tested the anti-proliferative activity of capsanthin with human triple-negative breast cancer (TNBC) and found that cell proliferation was inhibited after 24, 48 and 72 h of treatment. We also investigated the cellular and molecular mechanisms of the antitumor efficacy of capsanthin on TNBC cells and found that capsanthin delayed cell-cycle progression at the G1/S stage, that cyclin A expression was suppressed, and that p21 expression was upregulated. Capsanthin also inhibited the EZH2 expression and EZH2 could binding to the p21 promoter in TNBC cells. We further discovered that capsanthin has synthetic effects when combined with erlotinib (Tarceva). In the animal experiment, we found that the capsanthin-induced inhibition of TNBC cell proliferation decreased the incidence of the initiation and growth of TNBC cell–derived tumors in mice. Our study reveals that capsanthin exerted antitumor effects through delaying cell-cycle progression, induces erlotinib-sensitivity and inhibits tumor progression by inhibiting EZH2/p21 axis, and capsanthin is a potential drug candidate for development of a safe and effective therapy against TNBCs, especially for TNBCs that have developed resistance to targeting therapy.

## INTRODUCTION

New cases of breast cancer, which is a heterogeneous type of malignant tumor, in women are found more often than any other type of cancer and are the leading cause of mortality for cancers that afflict women, with the majority of deaths attributable to tumor metastasis [1]. Different breast cancer subtypes have different prognoses and responses to therapy. Consequently, various biomolecular and gene-expression profiling techniques have been increasingly used to classify the different types of breast cancer to facilitate better prognoses and the evaluation of treatment options. Gene-expression analyses have defined four principal breast cancer subtypes, namely luminal A, luminal B, HER2/neu, and basal-like, each of which is thought to be derived from a different stage of malignant progression of mammary epithelial cancer cells [2–4].

One basal-like subtype is also known as triple-negative breast cancer (TNBC), which has been classified as a distinct molecular subtype because it has a unique response to chemotherapy and other tumor-specific targeting agents [5]. Approximately 10 to 15% of all breast carcinomas are of the TNBC subtype, and they constitute ~75 to 80% of all basal-like tumors [3, 6]. TNBCs are characterized by the lack of expression of the receptors for estrogen, progesterone and of the human epidermal growth factor receptor 2 (HER2) [1]. TNBCs are more aggressive than other breast tumor types and thus are more likely to metastasize, and they are more prone to relapse after standard chemotherapy treatment. Consequently, it is necessary to identify both the molecular signatures and signaling pathways to the malignancy of TNBCs [6].

**Figure 1:**
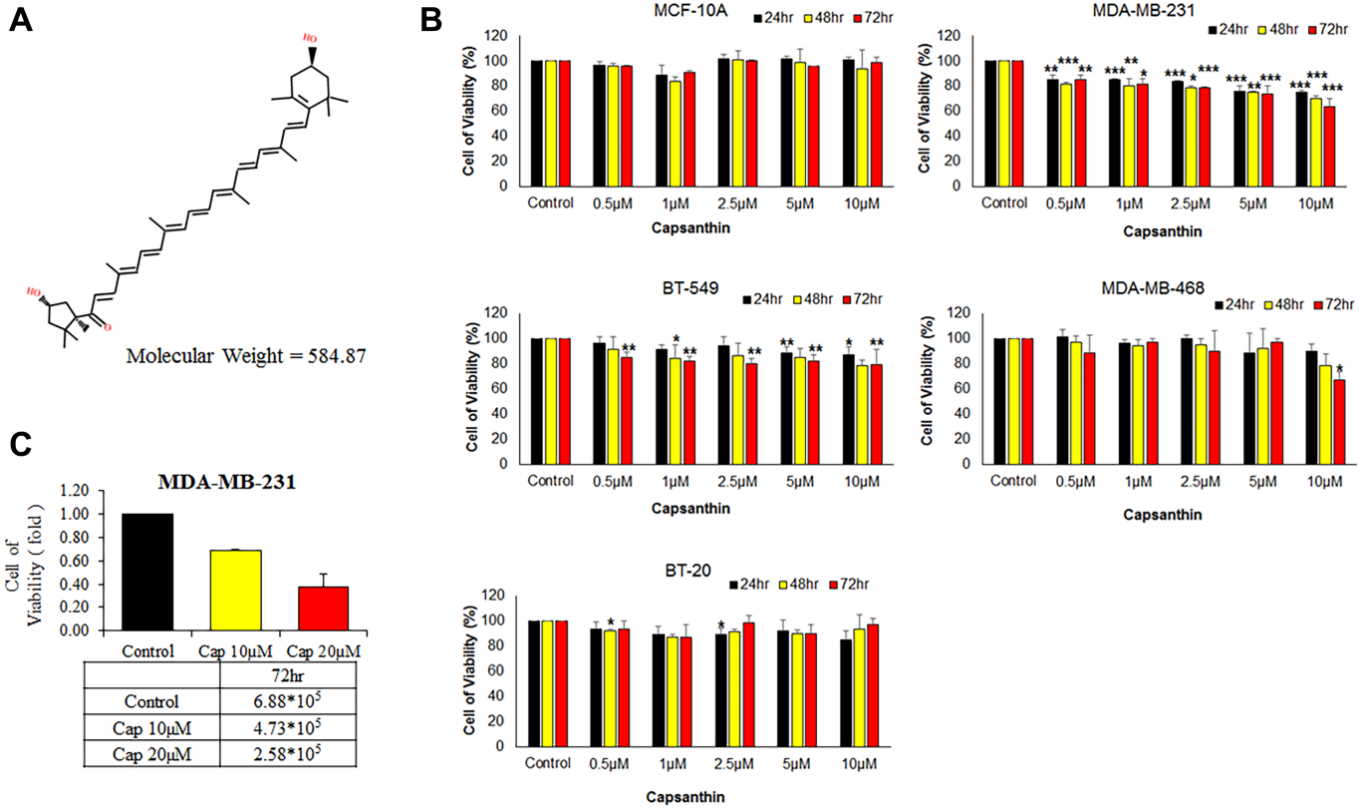
**Effects of capsanthin on the viability of different human breast cancer cell lines and normal human mammary epithelial cells**. (**A**) Chemical structure of capsanthin. (**B**) TNBC cells were treated with various concentrations of capsanthin for 24, 48 or 72 h and assessed for viability with the MTT assay. (**C**) Capsanthin inhibits the proliferation of MDA-MB-231 cells. The cells were treated with different concentrations of capsanthin for 72 h, after which the cells were counted. ^*^P < 0.05, ^**^P < 0.01, ^***^P < 0.001.

Current treatments for TNBC include surgery, radiation therapy, and chemotherapy [5, 7]. Powerful chemotherapies exist to fight many and varied cancers, but most have severe side effects including hair loss, nausea, and fatigue. Various naturally occurring carotenoids have proven to have anticancer and antioxidation activities, with certain of them showing a more effective anticancer activity than β-carotene, suggesting that they may be useful for cancer treatment. Capsanthin (Figure 1A) is found in the skin and meat of red peppers and belongs to the lutein class of carotenoids [8]. Capsanthin is a long-chain compound containing conjugated double bonds that can react with absorb free radicals to stably resonance. Importantly, capsanthin may have anticancer and antioxidant properties [9, 10].

EZH2 is a histone methyltransferase that contributes to the epigenetic silencing of target genes and regulates the cell lineage commitment and cancer progression [11–13]. Overexpression of EZH2 is often correlated with advanced stages of human cancer progression and poor prognosis including TNBC [12, 14]. Therefore, for the study reported herein, we characterized the molecular and cellular anticancer mechanisms of capsanthin using TNBC cells with the understanding that capsanthin may constitute a lead compound for the development of a drug that may target EZH2 and could improve the quality of life and/or reduce the chemotherapeutic toxicity of patients undergoing treatment for TNBCs [10, 11].

## RESULTS

### Effect of capsanthin on TNBC cell viability

We examined whether capsanthin is anti-proliferative activity to breast cancer cells in culture. The normal human mammary epithelial line (MCF-10A) and different TNBC cell lines (BT20, BT549, MDA-MB-468, MDA-MB-231) were initially cultured (3 × 10^3^ per 100 μL in 96-well plates) in a humidified atmosphere of 5% CO_2_ at 37°C for 12 h followed by incubation with capsanthin (0, 0.5, 1, 2.5, 5, and 10 μM) for 24, 48, and 72 h, with subsequent analysis of cell viability with the MTT assay (Figure 1B). Indeed, capsanthin significantly inhibited the viability of all the TNBC cell lines in a concentration-dependent manner but was non-effect towards MCF-10A cells (Figure 1B). Notably, MDA-MB-231 cells were more sensitive to capsanthin than the other breast cancer cells (Figure 1B). We also counted the number of cells remaining after treatment with 0, 10, or 20 μM capsanthin for 72 h. As we expected, the 72 h treatment decreased the viability of MDA-MB-231 cells (Figure 1C). These experiments demonstrated that capsanthin inhibits the proliferation of TNBC cells in culture.

### Capsanthin induces cell-cycle arrest at the G1/S phase in TNBC cells

**Figure 2:**
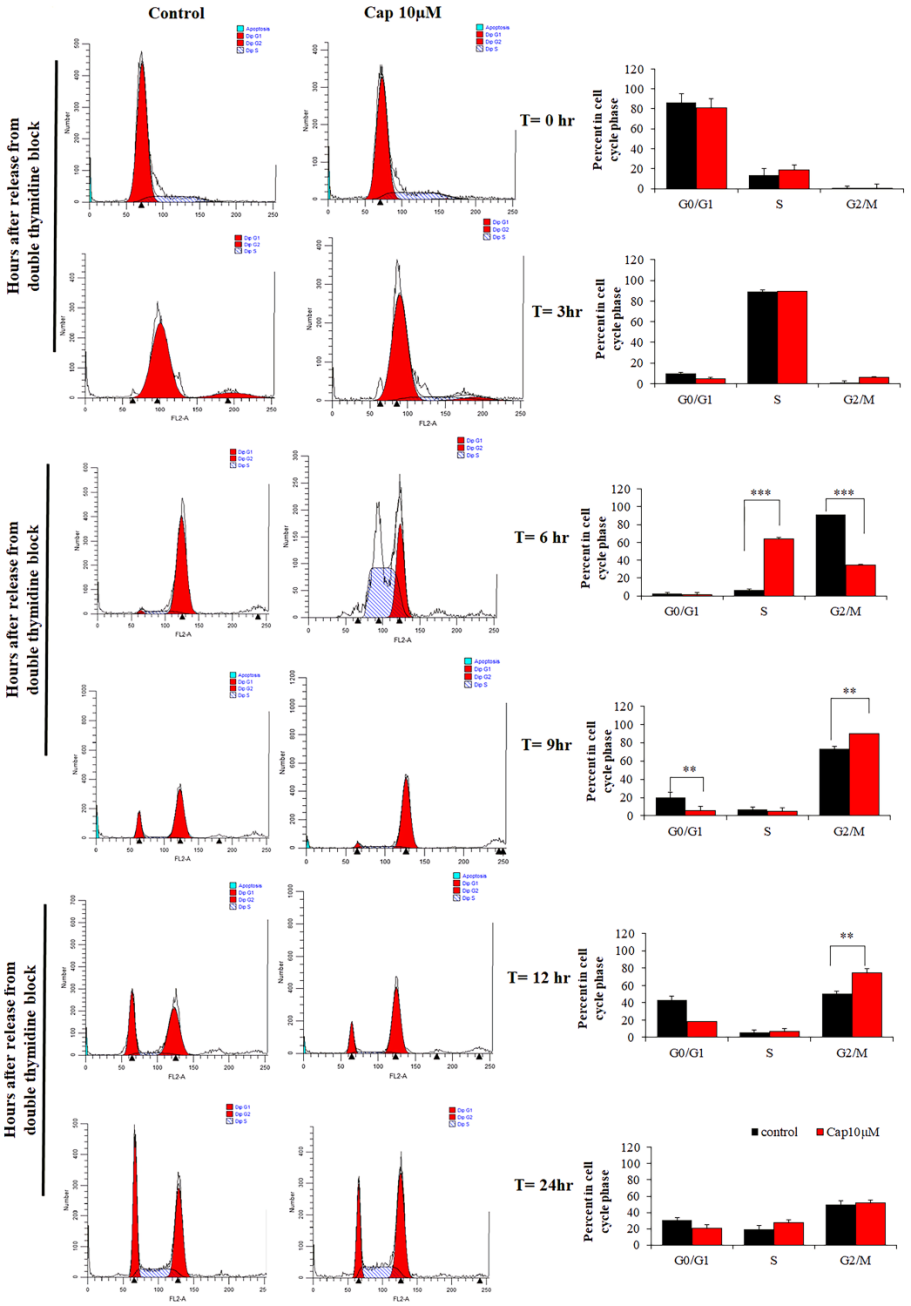
**Capsanthin delays cell-cycle progression in MDA-MB-231 cells.** MDA-MB-231 cells synchronized at G1/S were treated with 10 μM capsanthin for 0, 3, 6, 9, 12 or 24 h, after which their cell-cycle stage was assessed by flow cytometry. ^*^P < 0.05, ^**^P < 0.01, ^***^P < 0.001. (Cap, capsanthin).

Since the cell viability has been suppressed in TNBC cells. We were going to detected whether capsanthin influences cell cycle in TNBC cells by flow cytometer. The cell cycle is an essential role that cell could be proliferation and differentiation [8]. Thus, we postulated that the inhibitory effects of capsanthin on cell viability might be mediated by cell cycle. Therefore, the effect of capsanthin (10 μM) on the cell cycle was evaluated. In order to more accurately observe the effect of capsanthin on the cell cycle, we used a cell-synchronization approach with thymidine that determined whether capsanthin influences the cell cycle of MDA-MB-231 cells. We used a thymidine to further confirm that capsanthin arrested the cell cycle at G1/S phase. The MDA-MB-231 cells were synchronized using drugs and cells were harvested at 0, 3, 6, 9, 12, and 24 h after release from cell-cycle arrest and stained with propidium iodide to monitor the distribution of their cell-cycle phases. The results revealed that MDA-MB-231 cells became synchronized at late G1/S phase at 6 h after a double thymidine block followed by treatment with 10 μM capsanthin in fresh medium, demonstrating that capsanthin delays cell-cycle progression and induces G1/S phase arrest in MDA-MB-231. (Figure 2).

### Capsanthin suppresses EZH2-mediated epigenetic silencing of p21 in TNBC cells

**Figure 3:**
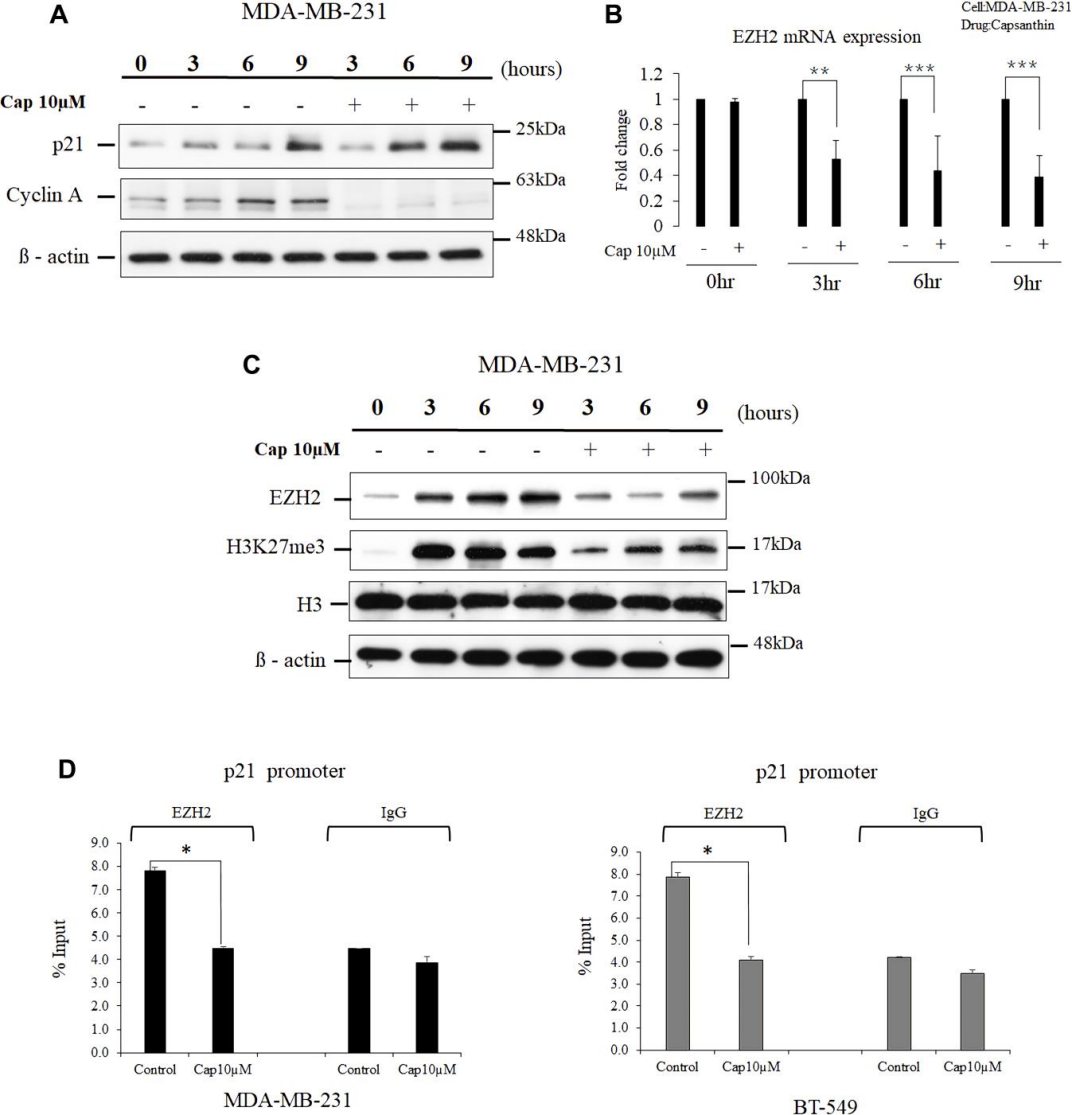
**Capsanthin decreases cellular EZH2 and suppresses the binding of p21 promoter by EZH2 in TNBC cells.** (**A**) Proteins were extracted from synchronized MDA-MB-231 cells at various times after treatment with 10 μM capsanthin for 0, 3, 6, 9, 12 or 24 h and then cell cycle–related proteins were analyzed with western blotting. (**B**) Synchronized MDA-MB-231 cells were treated with 10 μM capsanthin for 0, 3, 6, or 9 h, and qPCR was carried out to assess the cellular level of EZH2 mRNA. ^*^P < 0.05, ^**^P < 0.01, ^***^P < 0.001. (**C**) MDA-MB-231 cells were treated as described for panel A, and then proteins were extracted from the cells and subjected to western blotting for EZH2 and H3K27me3. (**D**) MDA-MB-231 and BT-549 with capsanthin downregulates EZH2 level and upregulates the p21 promoter. Capsanthin-treated cells were subjected to qChIP to assess the activation of the p21 promoter. ^*^P < 0.05. Cap, capsanthin.

Cell-cycle checkpoints ensure the proper execution of cell-cycle events. Because we found that capsanthin causes cell-cycle arrest at the G1/S stage in MDA-MB-231 cells, we postulated that it might influence the cellular levels of cyclin A and p21 (the cell-cycle checkpoint proteins for G1/S phase arrest). We checked the cellular levels of cell cycle-regulated proteins after capsanthin treatment. Therefore, MDA-MB-231 cells were treated a double thymidine block followed by treatment with 10 μM capsanthin in fresh medium. We then used western blotting to examine the cellular levels of both cyclin A and p21 in comparison with the control group at 0, 3, 6, and 9 h. Capsanthin decreased the level of cyclin A, whereas the level of p21 increased over the same period (Figure 3A).

EZH2 is a histone methyltransferase that promotes repression of the expression in certain genes and is the catalytic component of the polycomb repressive complex 2 [15]. It has been reported that EZH2 plays a central role in the regulation of cell proliferation and stem-cell differentiation. EZH2 is highly expressed in many malignant tumors including TNBCs, and elevated *EZH2* expression portends a poor prognosis for patients [12, 14, 16]. The progression of the cell cycle of cancer cells can inhibited upon inhibition of the EZH2 expression, and this results in upregulation of the cellular level of p21 [17–19]. Therefore, we surmised that p21 expression would increase upon treatment of cells with capsanthin, in concert with a decrease in EZH2. MDA-MB-231 cells were synchronized with thymidine and then treated with 10 μM capsanthin for 0, 3, 6, and 9 h. EZH2 mRNA level decreased at each of the 3, 6, and 9 h time points compared with the control in MDA-MB-231 cells (Figure 3B). The expression of EZH2 proteins and the cellular level of H3K27me3 marker were then measured with western blotting at 0, 3, 6, and 9 h post-stimulation, and the results were compared with those of the control group cells not supplemented with capsanthin. The results confirmed our hypothesis that capsanthin simultaneously decreases the expression of EZH2 proteins and the level of H3K27me3 (Figure 3C). These results indicated that capsanthin inhibits the production of both EZH2 protein and mRNA in TNBC cells (Figure 3B–3C).

We next assessed whether there was a epigenetic regulation between EZH2 and the p21 gene. The qChIP assessment revealed that an increase in EZH2 level led to its increased binding to the *p21* promoter, thereby inhibiting *p21* expression in both MDA-MB-231 and BT-549 cells (Figure 3D). These results demonstrated that capsanthin could downregulate EZH2 and increase the level of p21 via epigenetic regulation.

### Capsanthin has synthetic effects when combined with erlotinib (Tarceva) in MDA-MB-231 and MDA-MB-231-Tarecva resistant cells

**Figure 4:**
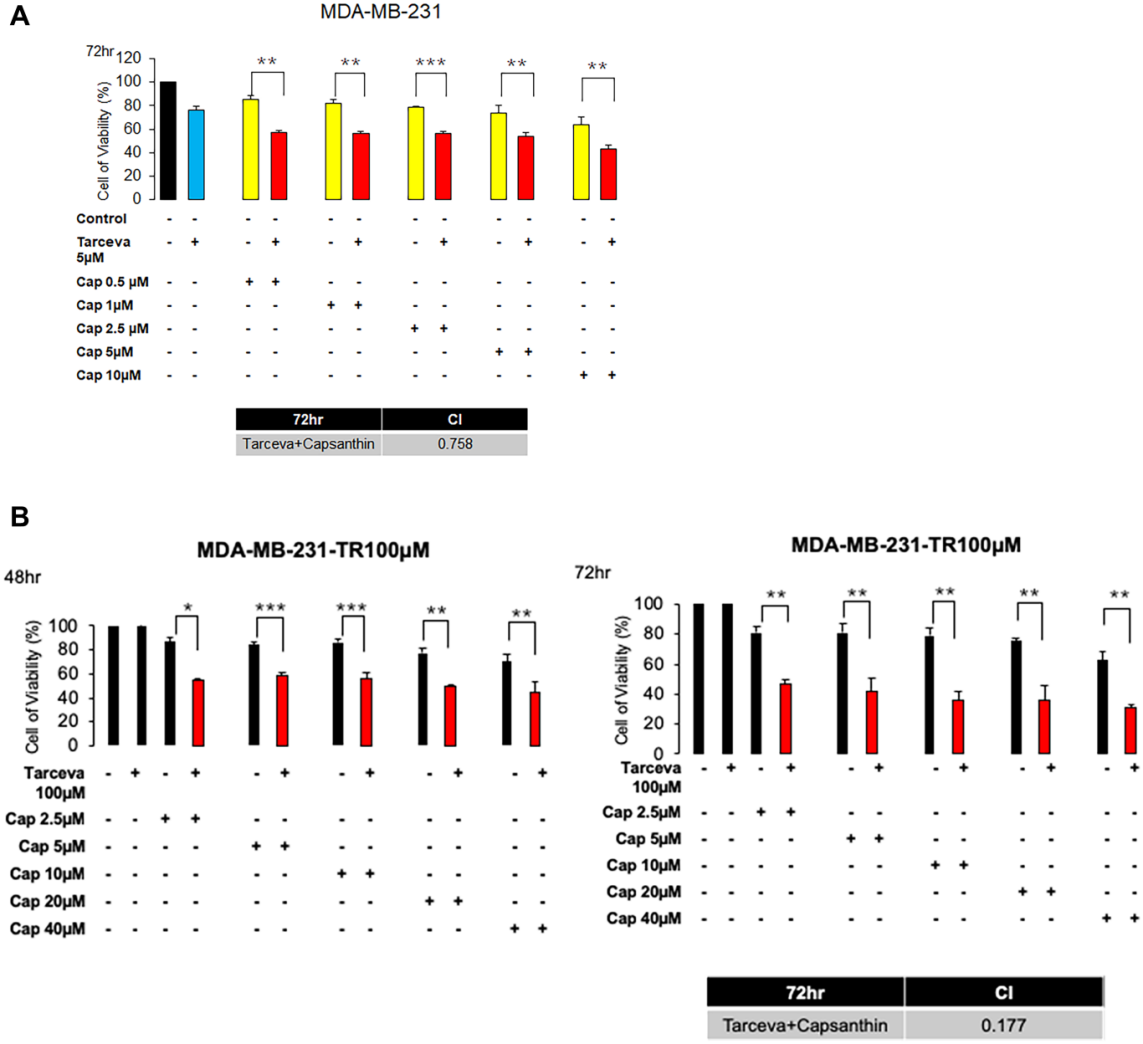
**Capsanthin has synthetic effects when combined with Tarceva in monotherapy and in two-drug combinations in MDA-MB-231 and MDA-MB-231-Tarecva resistant cells.** (**A**) MDA-MB-231 cell was treated with capsanthin(10 μM) and Tarceva(5 μM) in monotherapy and in two-drug combinations for 72 hours. ^*^P < 0.05, ^**^P < 0.01, ^***^P < 0.001. (**B**) MDA-MB-231-TR cell was treated with capsanthin (0, 2.5, 5, 10, 20, and 40 μM) and Tarceva(100 μM) in monotherapy and in two-drug combinations for 48 and 72 hours. ^*^P < 0.05, ^**^P < 0.01, ^***^P < 0.001.

Since we observed that capsanthin inhibits the G1/S phase of the cell cycle. And the chemotherapy drugs actually have serious side effects and we wonder whether capsanthin can reduce the chemotherapy concentration to avoid the damage of patient. Furthermore, we can only find genes with high expression levels for research or treatment, due to the lack of receptors for triple-negative breast cancer in target therapy. Some studies have suggested that the expression of epidermal growth factor (EGFR) is relatively high in triple negative breast cancer and it is the main cause growth. We used the EGFR-TKI (Epidermal growth factor receptor Tyrosine Kinase Inhibitor), called Tarceva in clinical. Therefore, we presume that whether capsanthin has synthetic effects when combined with Tarceva in MDA-MB-231 cells. MDA-MB-231 cells are treated with Capsanthin (0, 0.5, 1, 2.5, 5, 10 μM) and Tarceva (5 μM) in monotherapy or two-drugs combinations for 72 hours in MTT assay. We find that the synergized effects of Capsanthin with Tarceva in MDA-MB-231 cells are exceptionally great (the value of CI is 0.758, CI < 1) (Figure 4A). Next, it will often be produced drug resistance in target therapy. Therefore, we want to know whether capsanthin can restore the sensitivity of drug-resistant cells. We assessed the cell viability of capsanthin combined with Tarceva in Tarceva-resistant MDA-MB-231 cells by MTT assay. MDA-MB-231-TR (Tarceva-resistant) cells are treated with capsanthin (0, 2.5, 5, 10, 20, 40 μM) and Tarceva (100 μM) in monotherapy or two-drugs combinations for 48 and 72 hours. We discover that the synergized effects of capsanthin with Tarceva in MDA-MB-231-TR (Tarceva resistant) cells (the value of CI is 0.177, CI < 1) (Figure 4B). Overall, these data suggested that capsanthin has synthetic effects when combined with TKI-drug in MDA-MB-231 and MDA-MB-231-TR cells.

### Capsanthin inhibits the formation and progression of MDA-MB-231 cell–derived tumors in mice

**Figure 5:**
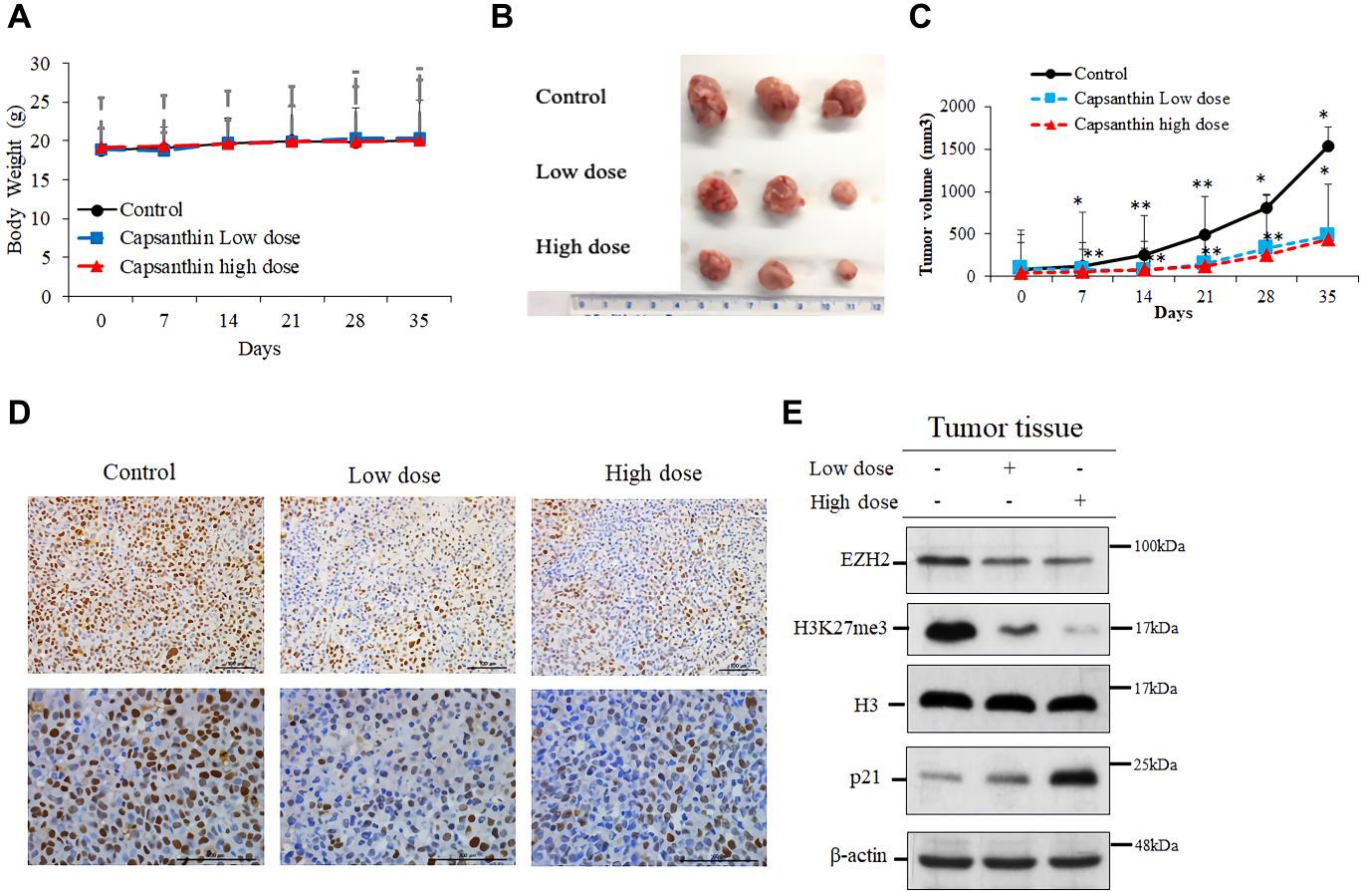
**Capsanthin inhibits the growth of MDA-MB-231 cell–derived tumors in mice.** (**A**) The tumor width (W) and length (L) were measured each week with calipers, and the tumor volume was calculated as L × W2 × 0.52. We injected capsanthin <low dose (0.036 mg/kg) and high dose (0.36 mg/kg)> into mice two times a week for 2 weeks, after which they were sacrificed. (**B**–**C**) Breast-cancer tumor growth was significantly inhibited in the groups treated with a low dose or high dose of capsanthin (*n* = 3). ^*^P < 0.05, ^**^P < 0.01. (**D**) Capsanthin inhibited the cellular level of EZH2 in both the low- and high-dose groups of TNBC tissues as assessed with immunohistochemistry (with anti-EZH2, brown color). (**E**) Capsanthin decreased the cellular levels of both EZH2 and H3K27me3 in tumor, whereas that of the p21 was increased.

To determine if capsanthin could inhibit the proliferation of TNBC cells *in vivo*, we injected MDA-MB-231 cells into mice. The time to tumor formation was 7–14 days for each experimental group. We injected capsanthin (low dose or high dose) into mice two times per week for 2 weeks. The tumor volume was calculated as L × W × 0.52. Compared with the control group (PBS), the mice groups injected with capsanthin either low dose or high dose had a significant delay in the onset of tumor formation and tumor volume on average (Figure 5B–5C) but not body weight (Figure 5A). To validate the EZH2 level in TNBC cells of excised tumors, tumor samples were analyzed with immunohistochemistry. EZH2 was downregulated in each of the low- and high-dose groups (Figure 5D). Then, we assessed the level of each of EZH2, H3K27me3 and p21 in tumors of mice of the control, low- and high-dose groups by western blotting. Capsanthin decreased the levels of EZH2 and H3K27me3, whereas it increased the p21 level (Figure 5E). In total, the results of all studies demonstrate that capsanthin plays an important role to inhibit the initiation and growth of MDA-MB-231 cell–derived tumors in mice by reducing the rate of tumor-cell proliferation.

## DISCUSSION

Breast cancer is the leading cause of cancer-related deaths in women worldwide. Gene-expression analyses have defined five breast cancer subtypes. Among these subtypes, we focused herein on TNBC, which is a malignant, heterogeneous disease [20]. Patients with TNBC have a poor prognosis, as their tumors tend to metastasize and an effective therapy is not available. Currently, the standard treatment options for patients with TNBC include surgery, chemotherapy, radiotherapy, and endocrine therapy, but these treatments have side effects that result in poor quality of life for patients [2, 21]. Therefore, we want to find a naturally occurring compound with anticancer activity that can be developed as a safe and effective drug, especially when a TNBC has developed resistance to other drugs. We therefore assessed the effects of capsanthin on TNBC cells. First, we determined that capsanthin inhibited proliferation of TNBC cells but had no effect on normal human mammary epithelial MCF-10A cells, which was an encouraging result as we wanted to find a treatment that would only target cancer cells. Next, we wanted to understand how capsanthin inhibits proliferation of TNBC cells because its associated mechanism has not been elucidated. We therefore examined the effect of the capsanthin-induced decrease in proliferation on the cell-cycle stages of TNBC cells because it is key to cell proliferation and differentiation. We used a double thymidine block to synchronize the MDA-MB-231 cells and found that capsanthin delayed cell-cycle progression and rapidly induced G1/S-phase arrest. We then demonstrated that the ability of capsanthin to cause G1/S phase arrest is related to a decrease in the cellular level of cyclin A, whereas the p21 level was increased as shown by western blotting.

Because it is unclear how capsanthin affects TNBC cells, however, we further investigated the molecular mechanism of capsanthin in TNBC cells. The multiprotein polycomb complex is an important mediator of transcriptional repression. EZH2, a polycomb group protein, is involved in cancer progression [22], and its expression provides an early marker of precancerous changes in histologically normal mammary tissue. The functional consequences of EZH2 overexpression may include promotion of differentiation and proliferation [23]. Many studies have shown that downregulation of p21 is associated with tumor differentiation, invasion, proliferation, and metastasis. p21 functions downstream of p53, which is a cyclin-dependent kinase inhibitor. Together, p21 and p53 constitute the G1-phase checkpoint because DNA damage cannot be restored without repair, which reduces the accumulation of damaged DNA prior to replication, and this inhibits tumor formation. Some reports have suggested that the downregulation of EZH2 level upregulates p21 level in cancer [18]. Therefore, we assessed whether capsanthin could affect EZH2 and p21 levels in TNBC cells. First, we demonstrated that capsanthin decreases the level of each of EZH2 mRNA and protein and increases the p21 expression in TNBC cells.

Whether the drug can have combined effect in the clinical. It has always been concerned. Therefore, we explore to the effect of capsanthin in combining TKI drug. Capsanthin has synergistic effect with Tarceva and inhibits the growth of cancer cell and resistant cancer cell (Figure 4). This part of the mechanism is explored to go again in the future.

**Figure 6:**
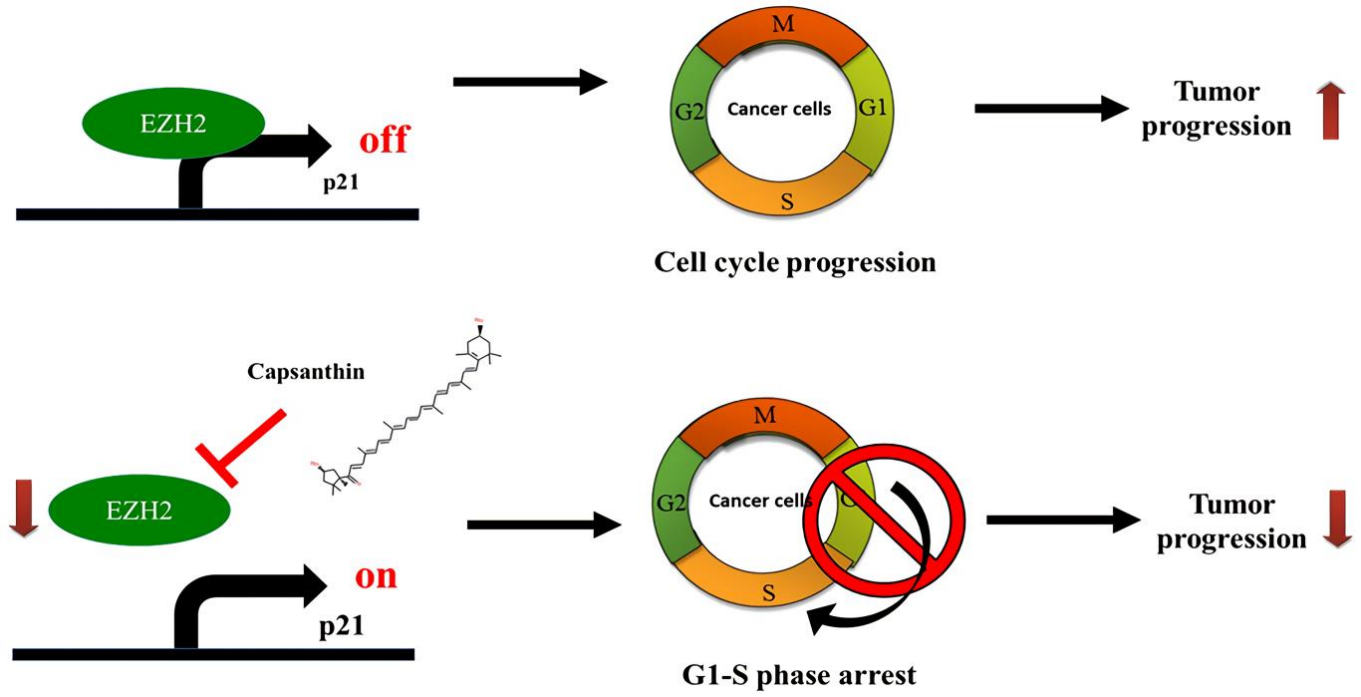
Schematic illustration depicting the roles of capsanthin in suppressing tumor progression of TNBC.

Next, we checked whether consistent results could be obtained from both animal and cell experiments. An immunohistochemistry analysis of tissues acquired in the animal experiments demonstrated that EZH2 level was downregulated in TNBC tissues from both the low- and high-dose capsanthin groups. We also demonstrated that capsanthin decreased the levels of both EZH2 and H3K27me3, whereas the p21 level increased. This suggests that EZH2-mediated epigenetically silencing p21 which may suppress by capsanthin-treated TNBC cells (Figure 6). It was previously reported that EZH2 and cancer metastasis are relevant [24]. We also want to further demonstrate whether capsanthin inhibits the metastasis of cells from TNBCs. Therefore, our results suggest that capsanthin may constitute a potentially efficacious drug for treatment of TNBCs via its ability to inhibit tumor progression.

## MATERIALS AND METHODS

### Cell lines, cell culture conditions, drugs and capsanthin stock solution

All cell lines used in this study were obtained from the American Type Culture Collection. The human TNBC (MDA-MB-231 and MDA-MB-468) cell lines were maintained in DMEM/F12 (1:1) with 10% (v/v) fetal bovine serum (GIBCO). The human TNBC lines BT-549 and BT-20 were maintained in RPMI-1640 supplemented with 10% (v/v) fetal bovine serum and 1% (w/v) penicillin/streptomycin. The non-tumorigenic mammary epithelial line MCF-10A was maintained in DMEM/F12 supplemented with 1.05 mM CaCl_2_, 100 ng/mL cholera toxin, 5% (v/v) horse serum (Gibco), 10 μg/mL insulin, 100 U/mL penicillin, 100 μg/mL streptomycin, 20 ng/mL EGF (Sigma), and 500 ng/mL hydrocortisone. All cells were maintained in a humidified incubator at 37°C under a 5% CO_2_ atmosphere. Capsanthin was dissolved in DMSO at a stock concentration of 60 mM and was stored at –20°C.

In order to establish the MDA-MB-231-Tarceva resistant cells, first, we cultured the cells in increasing doses of Tarceva (Roche) from 5 to 100 μM, which were increased stepwise over 6 months. We cloned the Tarceva-resistant cells after 8 months and keep the capsanthin concentration of 2 μM.

### MTT assay

We evaluated the ability of capsanthin to inhibit the proliferation of breast cancer cells and normal mammary cells using the MTT assay. In the wells of 96-well culture plates, MCF-10A, MDA-MB-231, BT-549 SKBR-3, and BT-20 cells were seeded at a density of 3 × 10^3^/well, and MCF-7 and MDA-MB-468 were seeded at a density of 5 × 10^3^/well and 8 × 10^3^/well, respectively. After treatment with or without capsanthin, the cells were cultivated for an additional 24 h at 37°C prior to the addition of 10% (w/v, final concentration) MTT in each well (Sigma; stock solution, 250 mg/mL in PBS). Following incubation for 2–4 h, the formazan crystals produced in each well were dissolved in DMSO (50μl/well), and the absorbance was measured at 570 nm in a Gen5 microplate reader (BioTeck). To quantify the relative viability of each cell line, its averaged absorbance was normalized to that of the corresponding control cells that had not been treated with capsanthin.

### Evaluation of combination effect

Liang *et al.* provide a method to evaluate the combination effect between two drugs, which is defined combination index (CI) [25]. The calculation formula for the evaluation is:

$$\text{CI}={\text{C}}_{\text{A},\text{x}}/{\text{IC}}_{\text{x},\text{A}}+{\text{C}}_{\text{B},\text{x}}/{\text{IC}}_{\text{x},\text{B}},$$

C_A,x_ and C_B,x_ refer to partial drug concentration of the combined use of A and B drugs to achieve x% effectiveness. IC_x,A_ and IC_x,B_ refer to the drug concentration when a single drug is used to reach x% effectiveness. The value of CI < 1 is considered to be a synergistic effect of two drugs. The value of CI = 1 is considered to be an additive effect. The value of CI > 1 is considered to be an antagonistic effect.

### Flow cytometry

Cell-cycle analysis was performed by directly staining DNA with propidium iodide (Sigma No. P4170). Cells growing in adherent cultures were trypsinized, washed with PBS, and fixed in 70% (v/v) cold ethanol overnight at –4°C. After removing the ethanol, 10^6^ cells were suspended in hypotonic fluorochrome PBS solution that contained 20 μg/mL propidium iodide, 0.2 mg/mL RNaseA (Invitrogen, No. 12091021), and 0.1% (v/v). Triton X-100 (Merck KgaA, Germany). The cells were then incubated at room temperature in the dark before analysis with flow cytometry (FACSCalibur, Becton-Dickinson). The cells were quantified with CellQuest software (Becton-Dickinson) and are reported as percentages.

### Cell-cycle synchronization by double thymidine block

Exponentially multiplying cells at a density of 5 × 10^5^/mL were diluted with fresh medium to 3.5 × 10^5^ cells/mL. Then, thymidine (50× stock solution in serum-free medium) was added into each cell culture at a final concentration of 2 mM. The cells were released from this first thymidine block by centrifugation at 600 × *g* for 5 min, carefully removing the supernatant, washing the cell pellet in 200 volumes of serum-free medium at 37°C, and then suspending the cells at a final density of 3.5 × 10^5^ cells/mL in fresh medium containing 24 μM deoxycytidine. After a 90-h release period, the second thymidine block was initiated by diluting the cell cultures with fresh medium so that the final cell density was 3.5 × 10^5^ cells/mL and then adding thymidine to a final concentration of 2 mM. The cells were released from the second thymidine block by centrifugation at 600 × *g* for 5 min and then suspending them at a density of 3.5 × 10^5^ cells/mL in fresh medium containing 24 μM deoxycytidine.

### Preparation of soluble cell extracts and western blotting

For preparation of soluble protein in cell lysates, cells were first washed twice with ice-cold PBS and sonicated in 20 mM Tris, pH 8.0, 150 mM NaCl, 1 mM EDTA, 0.5% (w/v), Nonidet P-40 with protease(s) and 25 mM NaF, 2 mM Na_3_VO_4_, 0.1 mM PMSF, and 20 μg/mL aprotinin. The soluble and insoluble fractions were prepared by centrifugation at 15,000 × *g* for 15 min at 4°C. The soluble proteins were separated by electrophoresis through SDS-polyacrylamide gels (6, 8, and 15% w/v acrylamide) and then electrophoretically transferred to a PVDF membrane. [Subsequently, each membrane was blocked with 5% (w/v) skim milk in Tris-buffered saline containing 0.1% (w/v), Tween-20 for 1 h at room temperature and then hybridized with the appropriate primary antibody (diluted in tris-buffered saline) with gentle agitation overnight at 4°C. After washing three times with this buffer, each membrane was incubated with an appropriate secondary antibody for 1 h at room temperature. Each immunopositive band was visualized with enhanced chemiluminescence detection (GE Healthcare). The following antibodies and chemicals were used: anti-EZH2 (1:1000; BD), anti-H3K27me3 (1:1000; Abcam), anti-histone H3 (1:1000; Santa Cruz Biotechnology), and anti-α-tubulin (1:5000; Sigma); MG-132 was from Millipore, and 3-deazaneplanocin A-HCl was from Cayman Chemical Company. Images were quantified with Image J software.

### Isolation and quantitative RT-PCR

Total RNA from cells was extracted with TRIzol reagent (Invitrogen). cDNA was synthesized from 2 μg total RNA in mixtures containing oligo dT primer, 10 mM dNTP, MML-V reverse transcriptase, RNase inhibitor, 0.1 M DTT and 5-fold first-strand buffer and incubated at 37°C for 50 min and then at 70°C for 15 min, and finally kept at 4°C. Quantitative real-time PCR (qPCR) was performed in mixtures containing 2 μL cDNA, 2.5 mM of a dNTP mixture, 2 μM of each specific primer, 1 U of Taq DNA polymerase, and a 10-fold concentrated reaction buffer. The reaction procedure was by denaturation at 94°C for 5 min, amplification at 94°C for 30 s, at 60°C for 30 s, and finally at 72°C for 30 s. A LightCycler 480 II system (Roche Applied Sciences) and reagents from a Fast Start DNA Master Plus SYBR Green I kit (Roche Applied Sciences) were used. The sequences of the specific primers for the following genes were: EZH2, forward, 5′-CAGTAAAAATGTGTCCTGCAAGAA-3′ and reverse, 5′-TCAAGGGATTTCCATTTCT sCTTTCGA-3′; GAPDH, forward, 5′-ACCACAGTCCATGCCATCAC-3′ and reverse, 5′-TCCACCACCCTGTTGCTGTA-3′. The mRNA transcribed from *EZH2* was normalized to that of GAPDH, which served as an internal control.

### Quantitative chromatin immunoprecipitation (qChIP) assay

Chromatin was isolated from MDA-MB-231 and BT-549 cells (treatment of capsanthin for 12 hr) using reagents of the ChIP assay kit (Millipore Inc.) and precipitated with the anti-EZH2. Immunoprecipitated DNA was PCR-amplified with primer sets covering specific regions of the p21 [26]. To amplify the DNA of each gene within a linear range, the quantitative real-time PCR (qPCR) procedure for serial dilutions of input DNA was: 94°C for 5 min, followed by 45 cycles of 94°C for 30 s, 60°C for 30 s, and 83°C for 30 s. Quantitative PCR was performed with the LightCycler 480 II system using Fast Start DNA Master Plus SYBR Green I kit reagents.

### Tumor orthotopic

Four-week-old female BALB/c nude mice were purchased from the Laboratory Animal Center, College of Medicine, National Taiwan University, Taipei, Taiwan. The animals were maintained under pathogen-free conditions. All animal care procedures followed the Institutional Animal Ethical Guidelines of the China Medical University. The each of nude mouse was orthotopically inoculated with 5 × 10^6^ tumor cells. Each group consisted of three mice. The tumor width (W) and length (L) were measured each week with calipers, and the tumor volume was calculated as L × W2 × 0.52. We injected capsanthin <low dose (0.036 mg/kg) and high dose (0.36 mg/kg)> into mice two times a week for 2 weeks, after which they were sacrificed.

### Immunohistochemistry

Immunohistochemical staining was performed on paraformaldehyde-fixed paraffin sections containing thin slices of the implanted tumors retrieved from capsanthin-treated and control mice after they were sacrificed. The tissues were stained with hematoxylin and eosin followed by incubation with anti-EZH2.

### Statistical analysis

Values are expressed as the mean ± SD and were analyzed using one-way analysis of variance followed by the Bonferroni post-hoc test to evaluate differences between multiple groups. All statistical analyses were performed using SPSS for Windows, Version 10 (SPSS, Inc.). A value of *p* < 0.05 was considered to reflect a statistically significant difference between experimental values.

## References

[1] Dawood S. Triple-negative breast cancer: epidemiology and management options. Drugs. 2010; 70:2247–58. 10.2165/11538150-000000000-00000 21080741

[2] Lehmann BD, Pietenpol JA. Identification and use of biomarkers in treatment strategies for triple-negative breast cancer subtypes. J Pathol. 2014; 232:142–50. 10.1002/path.4280 24114677 PMC4090031

[3] Aleskandarany MA, Green AR, Benhasouna AA, Barros FF, Neal K, Reis-Filho JS, Ellis IO, Rakha EA. Prognostic value of proliferation assay in the luminal, HER2-positive, and triple-negative biologic classes of breast cancer. Breast Cancer Res. 2012; 14:R3. 10.1186/bcr3084 22225836 PMC3496118

[4] Blenkiron C, Goldstein LD, Thorne NP, Spiteri I, Chin SF, Dunning MJ, Barbosa-Morais NL, Teschendorff AE, Green AR, Ellis IO, Tavaré S, Caldas C, Miska EA. MicroRNA expression profiling of human breast cancer identifies new markers of tumor subtype. Genome Biol. 2007; 8:R214. 10.1186/gb-2007-8-10-r214 17922911 PMC2246288

[5] Yagata H, Kajiura Y, Yamauchi H. Current strategy for triple-negative breast cancer: appropriate combination of surgery, radiation, and chemotherapy. Breast Cancer. 2011; 18:165–73. 10.1007/s12282-011-0254-9 21290263

[6] Neve RM, Chin K, Fridlyand J, Yeh J, Baehner FL, Fevr T, Clark L, Bayani N, Coppe JP, Tong F, Speed T, Spellman PT, DeVries S, et al. A collection of breast cancer cell lines for the study of functionally distinct cancer subtypes. Cancer Cell. 2006; 10:515–27. 10.1016/j.ccr.2006.10.008 17157791 PMC2730521

[7] Liedtke C, Mazouni C, Hess KR, André F, Tordai A, Mejia JA, Symmans WF, Gonzalez-Angulo AM, Hennessy B, Green M, Cristofanilli M, Hortobagyi GN, Pusztai L. Response to neoadjuvant therapy and long-term survival in patients with triple-negative breast cancer. J Clin Oncol. 2008; 26:1275–81. 10.1200/JCO.2007.14.4147 18250347

[8] Gloria NF, Soares N, Brand C, Oliveira FL, Borojevic R, Teodoro AJ. Lycopene and beta-carotene induce cell-cycle arrest and apoptosis in human breast cancer cell lines. Anticancer Res. 2014; 34:1377–86. 24596385

[9] Nishino H, Murakosh M, Ii T, Takemura M, Kuchide M, Kanazawa M, Mou XY, Wada S, Masuda M, Ohsaka Y, Yogosawa S, Satomi Y, Jinno K. Carotenoids in cancer chemoprevention. Cancer Metastasis Rev. 2002; 21:257–64. 10.1023/A:1021206826750 12549764

[10] Mori T, Ohnishi M, Komiyama M, Tsutsui A, Yabushita H, Okada H. Growth inhibitory effect of paradicsompaprika in cancer cell lines. Oncol Rep. 2002; 9:807–10. 12066213

[11] Chou RH, Yu YL, Hung MC. The roles of EZH2 in cell lineage commitment. Am J Transl Res. 2011; 3:243–50. 21654879 PMC3102568

[12] Chang CJ, Hung MC. The role of EZH2 in tumour progression. Br J Cancer. 2012; 106:243–47. 10.1038/bjc.2011.551 22187039 PMC3261672

[13] Chou RH, Chiu L, Yu YL, Shyu WC. The potential roles of EZH2 in regenerative medicine. Cell Transplant. 2015; 24:313–17. 10.3727/096368915X686823 25647295

[14] Chien YC, Liu LC, Ye HY, Wu JY, Yu YL. EZH2 promotes migration and invasion of triple-negative breast cancer cells via regulating TIMP2-MMP-2/-9 pathway. Am J Cancer Res. 2018; 8:422–34. 29636998 PMC5883093

[15] Hussein YR, Sood AK, Bandyopadhyay S, Albashiti B, Semaan A, Nahleh Z, Roh J, Han HD, Lopez-Berestein G, Ali-Fehmi R. Clinical and biological relevance of enhancer of zeste homolog 2 in triple-negative breast cancer. Hum Pathol. 2012; 43:1638–44. 10.1016/j.humpath.2011.12.004 22436627 PMC4194857

[16] Varambally S, Cao Q, Mani RS, Shankar S, Wang X, Ateeq B, Laxman B, Cao X, Jing X, Ramnarayanan K, Brenner JC, Yu J, Kim JH, et al. Genomic loss of microRNA-101 leads to overexpression of histone methyltransferase EZH2 in cancer. Science. 2008; 322:1695–99. 10.1126/science.1165395 19008416 PMC2684823

[17] Hubaux R, Thu KL, Coe BP, MacAulay C, Lam S, Lam WL. EZH2 promotes E2F-driven SCLC tumorigenesis through modulation of apoptosis and cell-cycle regulation. J Thorac Oncol. 2013; 8:1102–06. 10.1097/JTO.0b013e318298762f 23857401 PMC3713495

[18] Chen WM, Huang MD, Sun DP, Kong R, Xu TP, Xia R, Zhang EB, Shu YQ. Long intergenic non-coding RNA 00152 promotes tumor cell cycle progression by binding to EZH2 and repressing p15 and p21 in gastric cancer. Oncotarget. 2016; 7:9773–87. 10.18632/oncotarget.6949 26799422 PMC4891083

[19] Li Z, Wang Y, Qiu J, Li Q, Yuan C, Zhang W, Wang D, Ye J, Jiang H, Yang J, Cheng J. The polycomb group protein EZH2 is a novel therapeutic target in tongue cancer. Oncotarget. 2013; 4:2532–49. 10.18632/oncotarget.1503 24345883 PMC3926847

[20] Sorlie T, Tibshirani R, Parker J, Hastie T, Marron JS, Nobel A, Deng S, Johnsen H, Pesich R, Geisler S, Demeter J, Perou CM, Lønning PE, et al. Repeated observation of breast tumor subtypes in independent gene expression data sets. Proc Natl Acad Sci U S A. 2003; 100:8418–23. 10.1073/pnas.0932692100 12829800 PMC166244

[21] Ito-Kureha T, Koshikawa N, Yamamoto M, Semba K, Yamaguchi N, Yamamoto T, Seiki M, Inoue J. Tropomodulin 1 expression driven by NF-κB enhances breast cancer growth. Cancer Res. 2015; 75:62–72. 10.1158/0008-5472.CAN-13-3455 25398440

[22] Hock H. A complex Polycomb issue: the two faces of EZH2 in cancer. Genes Dev. 2012; 26:751–55. 10.1101/gad.191163.112 22508723 PMC3337450

[23] Varambally S, Dhanasekaran SM, Zhou M, Barrette TR, Kumar-Sinha C, Sanda MG, Ghosh D, Pienta KJ, Sewalt RG, Otte AP, Rubin MA, Chinnaiyan AM. The polycomb group protein EZH2 is involved in progression of prostate cancer. Nature. 2002; 419:624–29. 10.1038/nature01075 12374981

[24] Man S, Gao W, Zhang Y, Liu Z, Yan L, Huang L, Liu C. Formosanin C-inhibited pulmonary metastasis through repression of matrix metalloproteinases on mouse lung adenocarcinoma. Cancer Biol Ther. 2011; 11:592–98. 10.4161/cbt.11.6.14668 21304274

[25] Zhao L, Wientjes MG, Au JL. Evaluation of combination chemotherapy: integration of nonlinear regression, curve shift, isobologram, and combination index analyses. Clin Cancer Res. 2004; 10:7994–8004. 10.1158/1078-0432.CCR-04-1087 15585635

[26] Ma Z, Peng P, Zhou J, Hui B, Ji H, Wang J, Wang K. Long Non-Coding RNA SH3PXD2A-AS1 Promotes Cell Progression Partly Through Epigenetic Silencing P57 and KLF2 in Colorectal Cancer. Cell Physiol Biochem. 2018; 46:2197–214. 10.1159/000489589 29734178

